# Understanding the impact of the COVID-19 outbreak on vulnerable populations in Malaysia through an ethical lens: A study of NGOs and organizations involved in aid distribution

**DOI:** 10.12688/wellcomeopenres.17239.1

**Published:** 2021-10-12

**Authors:** Melati Nungsari, Chuah Hui Yin, Nicole Fong, Veena Pillai

**Affiliations:** 1 Sloan School of Management, Massachusetts Institute of Technology, Cambridge, USA; 2 Asia School of Business, Kuala Lumpur, Malaysia; 3 Diode Consultancy, Kuala Lumpur, Malaysia

**Keywords:** ethics, aid provision, humanitarian work, aid, public health, vulnerable group, policy making, government

## Abstract

**Background: **Globally, vulnerable populations have been disproportionately affected by the COVID-19 pandemic and subsequent responses, such as lockdown measures and mass vaccinations. Numerous ethical challenges have arisen at different levels, be it at the policy-making level or on the ground. For example, policymakers have to contain a highly contagious disease with high morbidity using scarce resources, while minimizing the medium- to long-term social and economic impacts induced by containment measures. This study explores the impact of COVID-19 on vulnerable populations in Malaysia by using an intersectional framework that accounts for overlapping forms of marginalization.

**Methods: **This study utilizes in-depth qualitative data obtained from 34 individuals and organizations to understand the impact of the COVID-19 outbreak on vulnerable populations in Malaysia. We utilize four principles of ethics to guide our coding and interpretation of the data – namely beneficence, non-maleficence, justice and autonomy. We utilize a frequency analysis to roughly understand the types of ethical issues that emerged. Using hermeneutic content analysis (HCA), we then explore how the principles interact with each other.

**Results: **Through the frequently analysis, we found that although beneficence was very prevalent in our dataset, so was a significant amount of harm – as perpetuated through injustice, the removal or lack of autonomy and maleficence. We also unearthed a worrying landscape of harm and deep systemic issues associated with a lack of support for vulnerable households – further exacerbated during the pandemic.

**Conclusions:** Policy recommendations for aid organizations and society to mitigate these ethical problems are presented, such as long overdue institutional reforms and stronger ethical practices rooted in human rights principles, which government agencies and aid providers can then use in the provision of aid to vulnerable populations.

## Introduction

Globally, vulnerable populations have been disproportionately affected by the COVID-19 pandemic and subsequent responses, such as lockdown measures and mass vaccinations. In Malaysia, approximately 100,086 cases (68% of total confirmed COVID-19 cases) from workplace clusters ^1^ were foreign nationals, the majority of whom were migrant workers working in the manufacturing sector ( *FMT*, 2021) – who were known to be a vulnerable population. The pandemic paralyzed the economy following the national lockdown (the Movement Control Order, MCO ^2^), adding another layer of difficulty to the existing precarity of stateless persons, refugees and asylum seekers, who do not have any legal documentation. Indigenous persons are also at risk due to their limited access to timely healthcare services in rural areas, compounded by disproportionately high poverty levels. Additionally, the resulting policy responses, particularly financial relief, discriminate against and exclude non-citizens. For those who are eligible, the lack of information and digital illiteracy has also restricted some of them from accessing help, despite the significant size of the recovery and stimulus programs launched by the government as of June 2021 (RM530 billion, or approximately USD125 billion) ( Prime Minister’s Office of Malaysia, 2021).

Over a year since the first MCO was imposed in March 2020 ( Prime Minister’s Office of Malaysia, 2020), Malaysia is still struggling to contain COVID-19, with cases reaching a record five-figure high in mid-July 2021. The void left by the state actors in providing support to vulnerable populations has led to the emergence of community movements. Among these is the grassroots #kitajagakita (“we take care of our own”), started after the first MCO was imposed, which has since become viral ( Savitha, 2020). The initiative provided a platform to connect those who wanted to offer help and those who needed help. Another common form of grassroots aid distribution has seen private funders donating funds or goods to existing civil society organizations (CSOs), such as non-governmental organizations (NGOs) and community-based organizations, which then deliver aid to the marginalized communities on their behalf.

However, numerous ethical challenges have arisen at different levels. Policymakers have to contain a highly contagious disease with high morbidity using scarce resources, while minimizing the medium- to long-term social and economic impacts induced by containment measures. Similarly, grassroots organizations also face similar ethical dilemmas on the ground, given their resource and capacity constraints. NGO workers and healthcare front liners often have to struggle with tough decisions, such as deciding who deserves help more. Vulnerable populations are most impacted by these ethical challenges due to pre-existing barriers to accessing healthcare, indeterminate immigration statuses and underlying, untreated health conditions. This study explores the impact of COVID-19 on vulnerable populations in Malaysia, including its migrant population, refugees and asylum seekers, stateless persons as well as indigenous people using an intersectional framework that accounts for overlapping forms of marginalization. Following that, we evaluate the responses towards the pandemic through an ethical lens, with the aim of providing recommendations to build better resilience and preparedness for future crises.

## Research background

### Disproportionate impacts of COVID-19 and lockdowns on vulnerable populations

COVID-19 was declared a pandemic by the WHO in March 2020, approximately three months after the first outbreak in China ( Ducharme, 2020). In its early stages, catchphrases like “the virus does not discriminate” or buzzwords like “a great equalizer” emerged in mainstream media, but we have since begun to realize that vulnerable populations are hit disproportionately hard. In this paper, we adopt the notion of vulnerable populations in public health by Frohlich & Potvin (2008), which refers to them as

… groups who, because of their position in the social strata, are commonly exposed to contextual conditions that distinguish them from the rest of the population. As a consequence, a vulnerable population’s distribution of risk exposure has a higher mean than that of the rest of the population.

Studies have found that racial and ethnic minorities faced disproportionate impacts ( Gaynor & Wilson, 2020; Tai *et al*., 2021), which may be traced to existing socioeconomic inequalities experienced by these minority populations. Many of them fall into the low-income group, and also lack adequate access to healthcare and social safety nets, compounded by exposure to higher infection risks given living and working conditions in which social distancing measures are often not feasible. Similarly, indigenous populations in Malaysia who predominantly live in rural areas have pre-existing socioeconomic disparities, such as disproportionately high poverty levels, and a lack of access to public services and infrastructure may reduce their capacity for responding to the crisis ( Verghis *et al*., 2020). 

Clark *et al.* (2020) have highlighted the vulnerabilities of immigrant populations in the United States of America (USA), particularly those who are undocumented or from the low-income group. In Malaysia, this was exacerbated by the surge of xenophobia as healthcare resources were stretched thin, causing many to feel threatened by the presence of foreigners. Heightened uncertainty coupled with increased nationalism in some countries may lead to an “us versus them” mentality ( Bieber, 2020; Esses & Hamilton, 2021). The hyper-precarity of unskilled migrant labour in the “Global North” ( Lewis *et al*., 2015) is also paralleled in Malaysia. Labour abuse, such as deplorable living and working conditions, is common due to the lack of protection of migrant workers ( Devadason & Meng, 2014), leading to local COVID-19 outbreaks at the world’s largest glove manufacturing plant, located in Malaysia, exposing exploitative labour practices and underscoring their vulnerabilities ( *BBC*, 2020). In addition, migrants – especially irregular migrants – also face the constant fear of arrest and deportation. The government has been conducting large scale raids specifically targeting irregular migrants, including refugees and asylum seekers, since May 2020 despite the high risk of COVID-19 outbreaks in overcrowded immigration detention facilities ( Chew, 2020; Wahab, 2020), having earlier justified their decision to push back boatloads of Rohingya asylum seekers as a preventative measure ( Yogendran, 2020). More recently, the government deported more than 1,000 Myanmar migrants and refugees in early 2021, in the midst of a military coup and the worsening pandemic in Myanmar, this time stressing the need to clear those overflowing immigration detention centers ( *BBC*, 2021), directly defying international law and the principle of non-refoulement ( *Reuters*, 2021).

Control measures aside, the debate on “lives versus livelihoods” remains a conundrum for policymakers. Malaysia has the highest stringency index in the Southeast Asian region (80.56) for its COVID-19 restrictions ( Hale *et al*., 2021), yet it also has the seventh highest number of daily new confirmed cases in the world (at 516 cases per million people as of August 1, 2021) ( Ritchie *et al*., 2021). The post-MCO economic slowdown has had a devastating impact on the labour market, with migrant workers often the first to be laid off under such circumstances. Additionally, particularly high-risk areas were placed under an “enhanced” lockdown, where individuals were not allowed to leave their homes and had food delivered to their doorsteps. These areas were disproportionately occupied by vulnerable groups, including undocumented and stateless individuals ( Povera & Harun, 2020).

Their precarity is exacerbated by the exclusion of non-citizens from any national economic recovery plan ( Nungsari *et al*., 2020; Sandanasamy *et al*., 2020). The stateless population is also one of the most neglected groups in policy responses, with their lack of legal documentation denying them the most basic rights, such as the right to employment, access to public services and the right to travel without fear of arrest. Many of them are living in chronic poverty, with a limited capacity to respond to the pandemic and economic crisis ( Allerton, 2017; Mohamed Razali, 2017).

### Ethical issues in COVID-19 responses

Due to market and state failures in providing COVID-19 relief to vulnerable populations, the void has been filled by grassroots organizations ( Tayeb & Hong, 2021). While studies have shown that such community-based efforts played an integral role in effective responses towards the pandemic ( Marston *et al*., 2020; Miao *et al*., 2021), there is a lack of understanding of the possible resulting ethical challenges. This is expected, given the urgency of the public health crisis, which may mean compromising on in-depth pre-assessments of ethical aspects to a certain extent. However, closer scrutiny through an ethical lens is essential in order to build better preparedness and resilience for any future crises.

In recent decades, the study of ethics has expanded beyond the philosophical realm to the practical application of ethical principles, especially in the medical and business fields. The four bioethics principles postulated by Beauchamp & Childress (1979), for instance, have become the cornerstone guiding medical practice all over the world. In the field of humanitarian and charity aid, some scholars have underscored the need to address ethical challenges arising from intervention on the ground ( Bell & Carens, 2004; Gasper, 1999; Schwartz *et al*., 2010), although the development of applied ethics in CSOs still lags behind the medical field. One of the very few guidelines available is the Code of Ethics and Conduct for NGOs, published by the World Association of NGOs in 2004 ( World Association of NGOs, 2004).

In this study, we adapt the aforementioned four fundamental principles of ethics, which are typically used in medical fields and in the clinical provision of health services ( Varkey, 2021), in order to study the provision of humanitarian aid and services during Malaysia’s public health crisis. Although we use the definition and essence of the principles as introduced and explored in the literature ( Beauchamp & Childress, 1979; Gillon, 1994b; Gillon, 1994a), the method by which we adapt them for the discussion of aid to vulnerable groups requires some explanation. In the following section, we present our versions of the definitions alongside specific examples from our unique setting to elucidate how the existing definitions were operationalized.

### Application of the four fundamental principles of ethics

The first principle is autonomy, whereby individuals and organizations have the power and ability to make rational decisions and moral choices, and are empowered to exercise their capacity to self-determination. Autonomy in the provision of aid can be seen in the following examples: a leading representative from a beneficiary group requests that specific items be included in food aid packages (perhaps because it is commonly used); or an NGO is empowered by external funders or by their own mission and internal funding to provide aid or additional services. A specific excerpt from the data is as follows:

So we asked them, okay, like, what's more convenient for you, and … most of them requested food baskets, and they also told us, “okay, this is what we want. We know that it's probably not something that you agree with, but this is what we eat here” … pretty much our role is just getting the funds, transferring the funds, buying food and delivering them. What kind of food they want, when to be delivered, most of those are decided by the community themselves.

The second principle is beneficence, where an individual or organization acts to benefit their beneficiaries and/or promote their welfare. For example, NGOs may organize cultural competency training for healthcare providers to better serve refugee and migrant communities, or organize capacity building programs for refugees in order to increase their employability in the job market. In the context of our dataset, we also found evidence of beneficence in NGO’s response to urgent need of shelter as seen below.

But the non-Malaysian[s] who … rent rooms. And a lot of the landlords because of the government policies, they were also threatening … to kick them out if they didn't pay the rent. Yeah. So that's why we had to, like really help out with rental for three to four months.

The third principle is non-maleficence – doing no harm. Specifically, non-maleficence is demonstrated when beneficiaries are not harmed as a result of the provision of services or the presence of the individuals and organizations providing aid. One example is when organizations do not send over staff or volunteers to do community work for fear of spreading the virus to beneficiaries, or take additional steps to ensure the safety of their beneficiaries when they come and collect food aid – such as implementing staggered pickup times to avoid crowds, or having pickup locations in quieter areas to avoid increased visibility (which heightens their risk of getting arrested). Risk mitigation can also be a form of non-maleficence, as demonstrated below.

Our mission is reaching out to people who are in unsafe places. So risk mitigation is a huge factor in whatever we do…. We need to ensure that it comes back to the work of keeping everyone safe, and we have many conversations around how we work around unsafe situations.

It is important to note that the two principles of beneficence and non-maleficence are distinct and separate:

for those circumstances in which we have or recognize no obligation of beneficence to others (as we still have an obligation not to harm them)…. thus, the traditional Hippocratic moral obligation of medicine is to provide net medical benefit to patients with minimal harm – that is, beneficence with non-maleficence. ( Gillon, 1986)

To concretely see the differences in our context, consider a NGO that works with the rural poor in the provision of food aid. One example of beneficence *with* non-maleficence is when NGO workers undergo strict COVID-19 testing protocols and work in small groups when providing food aid. Conversely, an example of beneficence *without* non-maleficence is when NGO workers come in large groups while providing aid and do not obey public health measures such as masking up during distribution.

The final ethical principle is justice, whereby individuals and organizations treat their beneficiaries equitably and fairly, while lessening their burden in accessing services or aid. Examples of this are where NGOs work together to ensure that there are no any discriminatory practices and that the maximal number of individuals is included in aid distribution, or in partnership with beneficiary communities to figure out what should be involved in food aid, where to drop off the food and the list of beneficiaries. An excerpt of this principle in action is as follows.

As long as the beneficiaries need help, we will ask them where, which area, and why…. We encourage them to come to our center to collect the food packs. We don’t check whether what they say is true or not. Because of COVID, we have loosened up on this.

## Methods

### Consent and ethics approval

Prior to conducting the study, we underwent two research ethics reviews – the Ethics Consultative Services for Marginalised Population (ECS-MP) at the University of Malaya (UM) in Kuala Lumpur and the WHO’s Research Ethics Review Committee (WHO ERC, Protocol ID number: CERC.0075) – with approval obtained on 15 ^th^ of February and 27 ^th^ of January 2021, respectively. Written informed consent was obtained from all participants before the interviews started, who were also informed of their agency in ending the interview at any time.

### Participant sampling and selection

Inclusion criteria were being above the age of 18, having the capacity to provide consent and belonging to one of the following categories:

- members of populations on the move (i.e., stateless persons, regular migrants, irregular migrants, refugees, asylum-seekers or undocumented persons);
- indigenous populations (i.e., Orang Asli populations in West Malaysia or Orang Asal populations in East Malaysia);
- members of CSOs or leaders who work with these populations; or
- employers of members of these populations.

Participants was selected through convenience sampling: information about the study and calls for participants were disseminated through CSOs, their related networks and researchers’ professional and institutional networks, with an emphasis on its voluntary nature. The first round of participants were approached via a mixture of email, social media, and messaging apps such as WhatsApp. The rest of the participants were snowballed from the first round of participants who shared with us their contacts with their consent and were contacted via the same methods stated above.

The target sample size was originally 40–45 participants, i.e., roughly 5–7 participants per each of the following vulnerable populations: populations of the move (i.e. stateless persons, regular migrants, irregular migrants, refugees and asylum-seekers, and undocumented persons), and indigenous populations (i.e. Orang Asli populations in West Malaysia and Orang Asal populations in East Malaysia). However, being under a strict lockdown and having to collect data remotely limited our reach and access to these communities. We approached 75 individuals in total, and ended up with 34 participants. The number of participants who declined to participate is 41. 34 of the individuals who declined to participate did not respond to the call for participation or had scheduling conflicts and so, were unable to participate. 5 of the individuals declined because they didn't feel like they had the right expertise for the study or did not want to participate because they had participated in similar studies beforehand. The remaining participants declined without giving a reason. It is important to note that since the data was collected during the lockdown, many organisations and individuals were still providing food aid to the communities and working on the ground, which could explain the lack of response. 

Out of the total of 34 participants who were identified and interviewed, 31 of whom were representatives of NGOs and businesses, and 3 were independent individuals from the targeted categories. A number of the NGO representatives were also leaders of their own (vulnerable) communities (see Table 1 for more complete details). For example, Participant 5 is from an NGO in Klang Valley (i.e., the Kuala Lumpur Metropolitan Area) that serves migrants, refugees and stateless persons through the provision of aid as well as outreach and livelihood programs. The recruitment of participants and snowballing ended when data saturation was achieved: namely, when the team started hearing similar themes or findings from different participants.

**Table 1.** Details of the interviewees.

Participant number	State	Community served or a part of	Type of organization
1	Sabah	Stateless, LGBTQ+, sex workers	NGO providing aid
2	Klang Valley	Stateless, indigenous, unaccompanied minor refugees, trafficked victims	NGO providing aid, case management, education, and community placement
3	Klang Valley	Refugees	Individual
4	Sarawak	Indigenous, women	NGO providing aid, case management
5	Klang Valley	Migrant, refugees, stateless	NGO providing aid, outreach programs, livelihood programs
6	Klang Valley	Refugees, migrants	NGO providing aid, case management
7	Klang Valley	Refugees	Individual
8	Klang Valley	Refugees	NGO providing aid, case management, outreach programs, livelihood programs
9	Sarawak	Indigenous, urban poor	NGO providing aid, case management
10	Klang Valley	Migrants, refugees, stateless	NGO providing aid, case management, outreach programs, livelihood programs
11	Sarawak	Indigenous persons, urban poor	NGO providing aid
12	Klang Valley	Migrants, refugees, stateless, women	NGO providing aid, case management, outreach programs, livelihood programs
13	Sarawak	Indigenous, rural poor, urban poor	NGO providing aid
14	Sarawak	Stateless, urban poor	NGO providing aid, case managements, legal aid
15	Sarawak	Indigenous persons, rural poor, women	NGO providing aid, outreach programs, livelihood programs
16	Klang Valley	Refugees	Business/social enterprise employing refugees
17	Klang Valley	Refugees	NGO providing aid, case management, education
18	Sabah	Stateless, indigenous	NGO providing aid, case management, education
19	Sabah	Stateless, indigenous	NGO providing aid, case management, education
20	Sabah	Stateless	NGO providing aid, case management, education
21	Klang Valley	Indigenous	NGO providing aid, case management, education
22	Klang Valley	Indigenous	NGO providing aid, case management, education
23	Klang Valley	Stateless	NGO providing aid, case management, education
24	Klang Valley	Indigenous	NGO providing aid
25	Sabah	Stateless, indigenous	NGO providing aid, outreach programs, education
26	Klang Valley	Stateless	NGO providing aid, outreach programs, education
27	Klang Valley	Refugees, migrants, urban poor	Business employing refugees
28	Klang Valley	Refugees	Individual
29	Penang	Refugees	NGO providing aid, access to medical care
30	Klang Valley	Migrants, refugees, stateless	NGO providing aid, access to medical care
31	Sabah	Stateless, indigenous	NGO providing aid, legal aid, case management
32	Klang Valley	Refugees	NGO providing aid
33	Klang Valley	Indigenous persons	NGO providing aid, outreach programs, case management
34	Klang Valley	Indigenous persons	Business providing aid and employment for indigenous persons

NGO = non-governmental organisation.

### Data collection and analysis

The interviews were conducted either in English, Bahasa Malaysia, Mandarin or combinations of these languages. Five of the 34 interviews were done over the phone and 29 through Zoom. All interviews were first recorded (both audio and visual, if the participant had turned on their cameras for the interview session). Then, the audio portion was extracted and transcribed verbatim. Finally, the transcript was translated to English, if needed. The interviews were between 45 and 75 minutes each, averaging 60 minutes in length, and conducted between February 15 ^th^ and March 26 ^th^, 2021. Data collection was led by N.F., the third co-author in the study. N.F. is a researcher-activist, cis-woman, trained economist with three years of research advocacy and human rights advocacy experience. No one else was present besides the researcher and participants in the interviews. The questions were developed based on all four researchers’ existing expertise and prior experience in the field – specifically, with interacting with members of vulnerable communities on the ground.

The transcripts were analysed using hermeneutic content analysis (HCA) ( Bergman, 2010); a three-step mixed methods tool. In the first step, a thematic analysis is conducted on the full dataset, the unit of analysis of which is a question-answer pair, with the full dataset containing 857 excerpts or data points. After cleaning the data and removing pairs that contained no information (such as small talk between the interviewer and interviewee), the dataset was reduced to 750 excerpts. The data was coded using Microsoft Excel (version 16.16.27), the HCA graph was produced using R (version 3.5.1), tables were produced in Microsoft Word (version 16.16.27), and other figures were produced using LucidChart. Deductive coding was done by imposing the four ethical principles outlined in the previous section onto the data. This was done by coding each of the 750 excerpts for both (affirmative) presences of the four principles, as well as their absences. Consider the following excerpt from the dataset, obtained from a participant who provided legal services to vulnerable groups. ^3^

I was trying to help a lady at the beginning of the lockdowns. That lady was actually from S [location]. Because I'm based in K [location a distance away from S], I could not travel because of MCO. I requested to the judge, I requested to the prosecution, I said I would like to represent her [in her court case] through Zoom.... The judge was willing to consider using Zoom but because at that time, the rules of court had not yet changed, there was no provision for such an application. The prosecution argued and actually objected to my request to have it heard over Zoom, even though that is the common sense approach at that time. In any event, I just prepared my submission, I filed my submission, I got another lawyer in S [location] to stand in for me. And I think at that time I felt that the government agency was really using this as an opportunity to oppress or to show how powerful they are. Other than that, when we were giving out aid, it was still okay because the police at the blockade ^4^ are quite okay with us. So when we passed by they allow us. So we didn't have a lot of difficulty there.

This excerpt was coded for the following principles – autonomy positive (i.e., existence of autonomy), autonomy negative (i.e., absence of autonomy), beneficence positive and beneficence negative. The specific parts of the excerpt that correspond to each principle are coded, using the excerpt above as an example. Note that some parts may contain multiple demonstrations or absences of ethical principles.

- Autonomy Positive – “ ***I*** requested to the judge, ***I requested*** to the prosecution, ***I said I would like to represent her*** through Zoom.”
- Autonomy Negative – “ ***The judge was willing to consider*** using Zoom but because at that time the rules of court have not changed, there was no provision for such an application. ***The prosecution argued and actually objected to my request to have it heard on Zoom*** even though that is the common sense approach at the time.”
- Beneficence Positive – “ ***I was trying to help a lady sat the beginning of the lockdown*** …I said ***I would like to represent her case*** through Zoom … In any event, ***I just prepared my submission, I filed my submission, I got another lawyer in S to stand in for me***.”
- Beneficence Negative – “ ***The prosecution*** argued and ***actually objected to my request to have it heard over Zoom*** even though that it is the ***common sense approach at that time***.”

The 34 transcripts were divided among three researchers who coded the data independently. To obtain a high and significant level of inter-rater reliability, we followed the coding process developed by MacQueen *et al.* (1998). First, the coders were assigned a small set of non-overlapping transcripts and independently coded them. Then the coders exchanged transcripts and re-coded that work to assess the consistency of code applications. Discussions were conducted to ensure that the coders were assessing the excerpts in a similar manner, and repeated until the code application was acceptable and consistent. After each group discussion, the coders continued coding more transcripts, and this process was repeated.

The second step of HCA is the plotting of the MDS graph to depict the relationships between the four principles of ethics highlighted above (i.e. maleficence, beneficence, justice, and autonomy in two valences each: positive and negative). We use the term “principles” and “themes” interchangeably. An MDS graph utilizes quantitative dimensional analysis to explore relationships between the themes to organize our data from the first step. Specifically, it visualizes the configuration of themes within a space by utilizing physical space between themes as a proxy for the degree of co-occurrence (i.e., similarity) of themes in the dataset ( Cox & Cox, 2008). The third step of the HCA is the recontextualization of the MDS graph using the primary dataset – that is, based on the relationships depicted in the MDS graph, we will use the transcripts obtained from our participants to explain or justify relationships depicted in the MDS graph. For example, if we find that principle X appears very close to principle Y on the MDS graph, we explore, using the qualitative data, why exactly that is the case by finding excerpts that help explain how X could be related to Y.

## Results

### Frequency analysis for the four ethical principles

The frequency of occurrence of the four ethical principles is presented in Table 2 below. As illustrated, beneficence (positive) appeared in more than half of the samples, indicating that responses towards the pandemic (e.g., aid distribution) by most of the organizations interviewed are well intended. For example:

So far, nobody has requested for counselling. But we have had requests like, can you help us pay rent? Or can you help us pay bills, medication, and things like that. So all they needed to do is send us a message and we'll transfer money to them, or we give them cash. And then after they've made their payment, they just send us a receipt just so that [we can verify] they did use it for medication…. But it's not really mandatory … [although] they’ve always provided us with receipts.

It is also very clear that many NGOs were forced to expand their services to include more individuals from the close social circles of previous beneficiaries – for example, NGOs who provided educational services to children found themselves helping the parents and family members of the children with rent, healthcare expenses etc. The lockdown’s disproportionate effect on (typically male) earning members of households also enabled women, who were previously not working, to earn an income to help support their families. In the following excerpt, one NGOs notes this shift, and comments on how they hope it will persist even after the lockdown:

When the lockdown was reopened again, and people could start working, we started our center [again] quickly, and there were many women could earn and because their husband have no jobs, and they could support their family. They could support paying the rental. So I think it was amazing, it was great, because at least they have some sort of income … understand the value of it.

**Table 2.** The frequency of occurrence of each of the eight themes in the dataset. n=750.

Theme (positive [+] / negative [-])	Frequency	Percentage (%)
Beneficence (+)	441	58.80
Justice (-)	258	34.40
Autonomy (-)	242	32.27
Beneficence (-)	240	32.00
Autonomy (+)	239	31.87
Non-maleficence (+)	220	29.33
Non-maleficence (-)	182	24.27
Justice (+)	166	22.13

However, the high frequency of justice (negative) also suggested that some beneficiaries were not treated fairly in these responses, such as the inability to reach all at-risk populations due to capacity and resource constraints faced by the organizations. In addition, the MCOs have also restricted their mobility in rural areas, where many of these populations live. Some beneficiaries have also mentioned instances of discrimination due to nationality, gender or sexuality.

There are a few NGOs who are delivering food … I choose to believe the community when they say that when the NGOs come in, they get everyone's information – their name, contact number, IC number. But the moment they [the stateless, trans individuals and sex workers] tell them that they don't have IC, no birth certificate, they still take their phone number and name, but the day the food aid is delivered, they don't get any. So I would say the organizations don't really pay attention to them. And even if there are police coming into the village, they don't really pay attention to the areas where the undocumented communities are located within the village.

Vulnerable groups, who already had limited mobility, were further marginalized.

So two weeks ago, we found out that there is one trans person who is outside of the area we're supporting. She had an accident. And so I think half her body is paralyzed, and she has no phone, there's no way to contact her. She can't speak anymore. But one of our trans friends was heading to the city and found her at a bus stop. And that's how we found out about her … So, I would say the biggest [part of] the community that we haven't been able to reach is those who don't have phones, and the disabled. It has been really difficult.

Autonomy was also an important emergent theme. The frequencies of both positive and negative autonomy were almost the same. In some cases, the participants had some agency, like choosing their beneficiaries or the aid delivery approach. However, their actions were also constrained by factors like mobility restrictions during lockdowns and the bureaucracy. For the beneficiaries, their autonomy is generally lower than that of organizations. One of the most common examples was that donors often made decisions about the contents of food packages without consulting the beneficiaries about what kind of food they actually needed.

Some people also tried to be really nice in the lockdown. They tried to bring food packets, they asked us to go and distribute, they come with us to distribute, they take pictures, and then, you know, it’s like they don't understand the needs of the people. And we needed to help people, but it's became too much for us … like we are a platform to fulfil their wishes…. They don't treat us as a platform where we build community. So they tried to listen to themselves and they follow the rules, but not ours, you know?

Evaluations of possible risks and damage in any intervention is important. While all NGOs and businesses interviewed tried to provide relief and assistance to vulnerable populations, some may have overlooked any possible harm caused, as reflected in 24.27% of excerpts showing non-maleficence (negative) themes. As shown below, interventions that do not consider local culture have led to discontent in the recipient community, which might consequently have negative repercussions for stateless persons. There was also evidence of non-maleficence, where safety precautions were taken when delivering aid to the beneficiaries. Practices such as social distancing, wearing masks and frequent sanitization were observed by the organizations in order to prevent possible infection.

For example, the biggest issue that happened back then, at some point they [medical NGOs] went and used the mosque as a place for COVID-19 testing. The locals were very angry of course because that place is a holy place and the stateless community don't really follow Islam. They follow what their ancestors followed, animism. So when the locals see their mosque being used for COVID testing and as a place to treat the stateless community. They feel very offended and insulted that their place has been polluted with this COVID-19 stuff.

### Multi-dimensional scaling (MDS) graph and recontextualization of data

**Figure 1.**
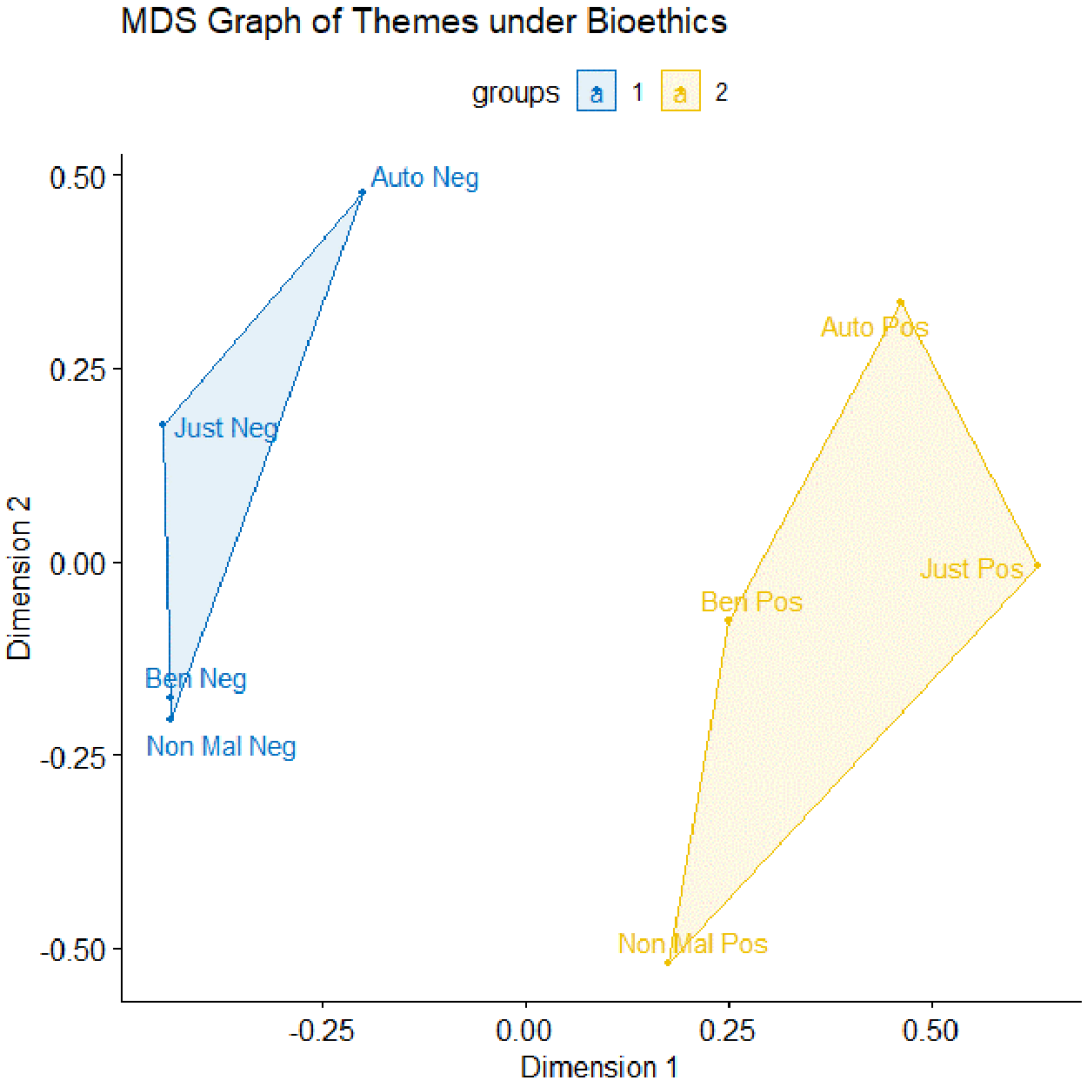
The multi-dimensional scaling (MDS) graph for the data, coded using the bioethics framework. n=750, clustering k=2, with a stress value of 0.026. Auto = autonomy; Just = justice; Ben = beneficence; Non Mal = non-maleficence; Neg = negative; Pos = positive.

In this section, we outline the findings from the second step of HCA by plotting out the eight themes above in relation to each other on a MDS graph. The units of analysis in this MDS graph were the question-answer pairs from the full set of interviews (n=750). Figure 1 depicts a clustering of k=2 with a low stress value of 0.026, indicating a high fit of the graph with our dataset.

We find two clusters – blue and yellow. The blue cluster depicts themes associated with negatively valenced themes (e.g., negative autonomy, negative justice, negative beneficence and negative non-maleficence). The yellow cluster depicts themes associated with positively valenced themes (e.g., positive autonomy, positive justice, positive beneficence and positive non-maleficence). The two separate clusters tell us that, overall, themes that are similarly valenced co-occur – i.e., positively valenced themes often occur together, and vice versa.

The positioning of the themes on the MDS graphs allows us to unearth an interesting relationship between negative beneficence and negative non-maleficence – that they co-occur almost all the time. To understand why this is the case, we proceed to the third step of HCA: the recontextualization of the relationship using interview data, which allows us to understand the relationship (i.e., do the themes simply co-occur, or does one theme cause another?) and mechanisms by which the themes relate to each other.

***Recontextualization by population.*** To do this, we first consider the full set of excerpts coded for both negative non-maleficence and negative beneficence. We then consider the affected population. Our respondents were either from or reported working with four broad populations, each with their own issues: migrants, refugees, stateless persons and indigenous persons. For example, migrants primarily reported being jailed as a result of immigration raids, navigating the legal system and being on the receiving end of hatred and xenophobia. The rest of the excerpts in this section were coded for both negative non-maleficence and negative beneficence.

Regarding deportation: we understand that many detainees can’t stay [in] detention conditions anymore and want to be deported. Some of them were kept away for many years, lost contact with their family members. So we totally understand that … we have to respect that. And we do respect that this is their decision. At the same time, we feel worry and concern[ed] because there's no Wi-Fi in the detention. So we're not sure whether they understand or they know about the current situation in their [home] country? We totally respect that decision to go back but the only questions we all have is “why now?” “Why in this situation?”
UNHCR [the United Nations High Commissioner for Refugees] has been denied access to detention and prison for a long time, almost … two years. So it's a very hard situation. It is difficult to find out about someone if they get arrested.… we totally lose contact with them. We don't know when is their court procedures ... we try to communicate, or we always ask our community members to tell us as much information as they have when one of them gets arrested. So through some friends or family members or some relatives, we will get information and we will start communicating with UNHCR and NGOs that can actually represent them. But for most of the time, it's not effective, because by the time we know where they are, it's already too late. They would be past court procedures, they've already been sentenced …
I mean physical conflicts between neighbours … I think they are what you can consider hate crimes. What happens online usually like hate speech, builds up and spills over into actual physical assaults, which are really unreported, unmonitored. I'm sure it's happening quite a lot. But also the community's trust in how we manage this case is also plays a role in them coming forward to report. If they don’t have proper access to justice, they don’t have the confidence of having legal representation. I mean, nobody will come forward, they know that Malaysia is a difficult place.

Refugees, on the other hand, talked more about issues of aid distribution and problematic relationships with NGOs and aid organizations.

Community organization[s] have to exist and it's very, very important. Without refugee communities, organizations like NGOs, UNHCR or other local NGOs cannot do their work. So they have to respect us, they have to understand … our work ethic, you know, they have to treat us as partner, not as you know, “I'm helping you, you just take what I need to give you. That's it, keep your mouth shut,” you know. We shouldn't have that kind of attitude, we should have more some empathy, you know, towards each other. And ask us what we need.

Refugees often suffer from “poverty porn”, in which external parties reach out to their communities and conduct one-time programs with significant media hype.

More newcomers and new people do it more because they want get more attention, like a big NGO … they don't care much, they will just take the credit, where they will take a picture of large group, and then they say, we have distributed this kind of food to this organization towards these people….
Okay, let me tell you this … they are doing good work. But I don't think it's enough…. And I feel like it's more of a show off than actually helping other people … in general, I think when you want to approach an NGO, you have to be careful when you're asking for help. I know for a fact that you have to have transparency, right. But taking pictures of the refugees that you helping and their conditions and their lifestyle and just posting them online, it's just so humiliating.
I know that they have donors, right? They are getting donations, and they want to inform their donors of what's happening, but don't do it in public. You know, for the people who are donating, you can create a newsletter or a group email … send emails, send updates, don't post pictures of those poor people and women and children online. It's just humiliating. I actually approached one of the NGOs to get help. Eventually, I didn't get any help. They suggested that I sell my car.

With that being said, refugees also faced similar issues as migrants in terms of law enforcement. A lot of anxiety and mental anguish was communicated through the interviews – particularly their inability to bribe the police and constant fears of being arrested.

I was driving here in KL with my friends, we went out. It was around 11pm. And there was a roadblock. And then the police officers, they stopped us. I didn't have my passport at the time because it was with my employee [ *sic*, employer], they were renewing my visa. So I did have a copy. And the copy has a stamp on it. My other friend was a student and she has a student visa. And the other two passengers. Two of them were refugees. They had a UN [United Nations] card. And when we were stopped, the police officer did not let us go until we paid money. And he told me, “I can take you down to the lockup for 24 hours and just hold you in a cell until we confirm that your passport is with your employer”. I'm like, “Okay, you can do it.” And he said he can make it easy for me…. there is a petrol station nearby with an ATM, you can go and withdraw there. Right? I went back to the car and I talked to my friends and I told them, let's collect whatever money we had. We had like RM300 and we paid him off. And he just let us go. Yeah. After we paid the money. He took my phone number.
I am absolutely terrified. When I see a police officer in uniform … you don't know when you're going to get stopped right? And I'm not saying all police officers are bad. I'm just saying, well, the majority, you know, they might stop you and ask for your documents. Even if you produce the documents, there is still possibility for them to ask for a bribe. And not only that, they will take your phone number and your address and they will contact you afterwards. To me it happened twice.

Stateless individuals mainly experienced a lack of access to services and trust in outsiders. For example, access to healthcare was still limited and communities had issues trusting external healthcare providers who were sent to collect samples for COVID-19 testing:

I was under a lot of pressure. Not just from the community, but the authorities. The locals didn't like that the health officers entered their community because the island had confirmed COVID-19 cases, they worried about how they were going to be viewed by other residents living near them. They felt it ruined their image, up to the point where they didn't want to trust the health officers – they thought [the officers] were lying to them. We worked together with the health officers because the community was not brave enough to go near the health officers. Even though we are not the ones who called the health officers … the hospital came to us to ask for help to assist them in COVID-19 testing. The hospital would come in everyday for testing. In the end, we were blamed [by the community] for this. But actually the hospital was also at fault.… [and t]he health officers who came by are not from the stateless community, some even are from Peninsular Malaysia who are very naive about stateless issues, their culture, traditions, beliefs…. Even though I have reminded them about these things, the hospital still doesn't listen. So when there are issues, I am answerable to [the community]. So in the end, I stopped focusing so much on the health of the community because why would I make myself go crazy trying to help the health officers who don't want to listen to me? To them, I'm just some village teacher and not a doctor or anything. So that's why I focus more on the welfare component…. I don't need to trouble myself and make the situation worse by trying to offer expertise in the health area. That's what I mean. I don't want to overstep my boundary with the hospital. Because the hospital is so very hated by the community there. So I don't want to handle these type of issues, why would I want to help people [the community] who don't want to help themselves? It's better if I just focus on my own work. So that's why in the end I was less focused on the health aspect of the crisis.

Stateless individuals could not access many aid packages and help either, due to their lack of citizenship.

During the MCO, a few of us we went around to check on people who actually needed help. We thought the government initiatives were very good and encouraging. But those people who are getting these kind of benefits are only Malaysians who have citizenship. So our focus at that time was actually to help the people who do not have citizenship. People who are not even looked upon at by any of these government agencies. Many of them are daily workers and they don't have wages per month. So the employer doesn't pay them if they don't work. But during the period also they couldn't work and a lot of them can't really afford pay for food and things like that. So we saw a lot of these kinds of situation, we saw a lot of these families who are actually having this kind of difficulty…. I actually met a whole family who had no identity cards … because one of the grandparents or parents did not do a proper registration of the marriage and register their identities. So they were stuck when they came to see us. And when I heard their problems, they started crying and they were really going through a really tough life. I met an Indonesian lady who married a Malaysian. But they got married under Syariah court in Sabah. But then the husband just ran away and never came back. And eventually divorced her through long-distance divorce…. and because of that she is considered as an immigrant in Malaysia and the son is actually an immigrant as well. So she couldn't work, but the son managed to enter a school. But then they have to pay for the textbooks and things like that. The principal was very kind to actually accept the son into the school. But the principal also requested them to apply for citizenship as well.
My first advice is specific to the government. Our government doesn't do research, they just get advice or they just do what they think is right…. So the impact from this action is that there are more marginalized communities who are not helped by this government…. Let’s say in this one village, there are 50 houses, and 20 houses from them are undocumented. So is it fair that just 30 houses will get the food pack[s]? … The second [impact] is because they didn't do research, the government doesn't have an exit plan…. This is publicly [known] and in all aspects – in education, welfare, economy. And as you can see, the last federal budget last November [2020] … was like a regular one, like the era before this pandemic. It is not suitable at all. So that's because they didn't do their research properly. They don't refer to NGOs or the correct persons who are working with the ground…. That's how loopholes happen in our policymaking and in their actions.

Finally, we considered the experiences of indigenous persons. It was very clearly that this population’s experience was very distinct from the other three groups, where misconduct and maleficence stemmed from their lack of legal rights and recognition in this country. Indigenous persons, however, are mainly recognized as legal citizens, and carry identity cards to prove so. ^5^ Indigenous persons even have a dedicated government agency created specifically to aid their communities and development. ^6^ Their experiences with non-beneficence and maleficence revolved around difficulties in accessing their legal right to aid and issues with NGOs or aid providers. For example, because indigenous persons often occupy rural spaces and there were limited coordinated efforts by the government to target these groups, many missed out on opportunities created to help struggling individuals like themselves.

You know the government cash aid program – they give you 200 ringgit in cash aid, right? So a lot of indigenous people, they didn't manage to get it… They need to pay for things like for the kids’ school and all that…. [but] because they don't stay in a place where there's any [Internet] reception so they missed the date to register.
If you remember, the government came up with an aid program of about RM500–1,000, which was a very good program, very helpful, but many indigenous people were not registered…. because they don’t pay income taxes or they don’t work, and so on. So we moved from food distribution to registration [so that they can qualify for cash aid]. The government was giving about 1,000 to one family and we were giving out at most 3,000 per village…. The problem is that they are entitled to it but they’re not getting it because of all this bureaucracy.

The aid providers also faced difficulties with local authorities and the various government agencies designed to aid indigenous groups.

We attempted to send aid in this area and there was this officer named Mr X. He was supposed to come up with letters [for] the police…. This was so that when we cross the boundaries between states, when there’s a roadblock, the police already [have] the letter instead of us having to explain that we have a letter. So we came in with two huge lorries and three or four tons of food in each. But Mr X gave us the wrong location! … The only response that we get from Mr X is “Sorry, sent you the wrong location.” … it took a few hours to get to that location intended to and when we were there, the local authorities were already there. Mr X … was suddenly trying to lead the operation. So apparently, he'd already prepared with some few cameras for show, to show on the agency’s Facebook page that they’re actually doing their job. But they didn’t, they didn’t even provide a police letter for us. So, two of our members got detained at a police roadblock. We had to call local politicians to sort things out for us. It was really a situation where everyone was really angry, because we were working on a tight schedule. We had to pay for the lorries – it wasn’t free.
When we were trying to get statistics from [the government agency], it was really annoying. They said they did not want to release statistics because of privacy and confidentiality, and that they wanted to protect survivors. But the numbers don’t harm anyone, they are just [the] reality of what is happening – that was our explanation. We’ve tried to have a good relationship with them, but at the same time, it is frustrating.

In many instances, indigenous persons were negatively impacted by ever-changing, non-targeted and unhelpful government policies. Aid was not customized for their needs, which meant that it was not impactful. For example, an aid provider reported the following.

The food basket also didn't take into account [the] number of things which the indigenous people wanted. For example, some of them wanted infant formula. Some wanted fresh food, like onions, but these were not in the basket because these are perishables.

The government also seemed to not have a sense of how many indigenous communities actually existed, highlighting the communications gap between leaders of said communities and the agencies tasked with helping them.

We have many cases of government aid given to villages where there are supposed to be 155 families but the government only supplies 150 food baskets. We don’t deal with these kinds of issues because that responsibility is the community’s. More important, it shows the community that they need to work together, they can work together, and they have leaders among them.

Aid providers also reported multiple difficulties in contacting said agencies, pointing out that many indigenous persons also faced difficulties in leaving their villages, with some getting harassed at roadblocks.

There was a newspaper article about how cases of domestic violence dropped in our area during the lockdown. [The government agency] were being congratulated. But we said no, you never picked up your phone, you never did you work [and] that’s why you think there are no cases even though there were a lot of cases going on.
Yes they were detained for buying food in large quantities. They weren't brought to the [police] station but there was still no reason to detain people who are on the roads in the middle of buying food…. They took down their names, their ID numbers….

**Figure 2.**
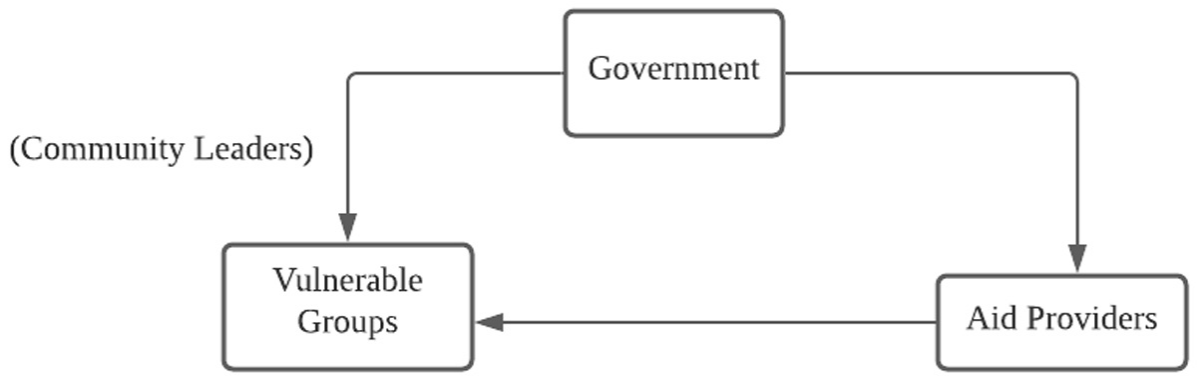
The circle representing the main actors of maleficence and non-beneficence during the pandemic.

***Actors and modes of maleficence and non-beneficence.*** To summarize the relevant data, we mapped out the parties that inflicted harm on vulnerable groups in the provision of aid, and the modes by which harm was inflicted by these parties. The results from this section are depicted in Figure 2 and Table 3 below.

**Table 3.** A breakdown of governmental actors reportedly involved in maleficence and non-beneficence.

Relevant governmental agents
Immigration officers and the system Legal system The police Detention center officers Healthcare system Agencies designed to help vulnerable groups Policies Politicians

Figure 2 shows a circle of maleficence and non-beneficence, including the main relevant actors. The top of the circle is the government and its various agents, such as immigration officers, the legal system, the police and policies created by parties in power (see Table 3). Some examples of harm include abuse in detention centres as a result of the aforementioned immigration raids.

So yeah, the lockup at [redacted], right. And the holding cells, they are divided into two sections, men and women…. And it was too small. It was like three meters by two meters, right? And one … bathroom and a toilet at the same time…. in front of each holding cell there was a CCTV [closed-circuit television] camera. And there was an operation room or a monitor room where they can watch everything that you do so even if you're taking a bath, they can see you naked. And this monitoring room it was for female and male officers at the same time. The place reeked with bad smell, it like smells like rotting flesh or rotten fish…. It's so dirty, we were not allowed to wear shoes or slippers so we were barefoot all the time. The holding cells, it's concrete … we were not provided with masks or blankets or anything. They just gave us these clothes, the colour was purple…and once you get inside they strip you of everything. And they search you like they search your body. They ask you to squat to cough. And I'm sorry to say that they kinda do searches in private areas. And the worst part that I remember is that the police officers, the male police officers, they were not allowed to go to the female holding cells, but you could see them all the time there…. They would watch us while we sleep. Right? And they will ask stupid questions. We had two Indonesian girls, they were held up with us. And he was just asking the girl like do you do massage, you know? So they would allow us to get out of the holding cell twice a day for food. And … the male police officer, asked the Indonesian girl to give him massage behind the bars. … The food is horrible. You don't know what they are giving you – is it coffee, is [it] tea, is it mix of two? You feel the grains of sand inside the liquid or the drink. The rice, the rice is so sticky that if you hit it on a wall, it will just stick right. And I have an allergy to seafood and they were serving fish day and night all the time. And that's it. It's the same meal. You have it twice a day. And the water. The water they gave us the water inside plastic bags.… twice a day. And then the amount … is not enough for a human being and the water tasted funny. Later on one of the girls that been inside for a longer time – I don't know if it's if this is true or not – she said that they put something inside the water to make people calm like so people don't make a fuss. So I really don't know.... We just slept a lot.

Through its actors, the government inflicted maleficence and non-beneficence in a few different ways. The first was by directly inflicting harm on vulnerable groups, sometimes through community leaders within these groups. This was seen in how aid distributed by the government on the ground was sometimes rationed by community leaders in unfair ways.

I don't really know the dynamics in the villages. But we heard that the community leaders, they have their own favourites, or they have their own lists of people. Of course I'm sure they are good community leaders out there. But we heard from those who complained that they didn't get the aid. They said the community leader didn't include them. They already have certain people who they want to give the aid to.

The government also inflicted harm through the fact that many core, important services during the pandemic were left unmanned, which left many vulnerable groups with nowhere to seek help from:

We have had many survivors coming to us for counselling … we asked if they have reached out to the [government agency] for help. And they said, yes, the hotline is apparently 24 hours, but no one ever picks up.... We try to help them through whatever they need from us. [see also the excerpt in “Recontextualization by Population” above]

The government unfairly politicized their work and rationed aid, as well as pushing the responsibility for the provision of aid and dealing with vulnerable groups to humanitarian organizations. These aid providers also faced tremendous difficulties in physically accessing the communities that they were trying to help, and were often placed as a disadvantage due to limitations imposed by the government, as a representative of an NGO explains.

The government gave a lot of aid during this time…. But sometimes … you'll be wondering how much money was actually used for these kind of packages. Because a lot of the food that is given by the government is way less than what we [are] giving. And then how do they determine who to give to? As I know the [the government agency] actually has a list of people who need help. But why are these things … given through political parties? … I'm pretty sure that some of these things don't get distributed and it is actually taken by some of their own family members as well. So I think that part is very unethical.… You are blurring the line between political parties and the government agencies.

Some aid providers also harmed vulnerable groups through their actions. One clear example that was often cited was lackadaisical physical aid distribution, which sometimes placed vulnerable groups at an increased risk for physical harm.

We noticed many NGOs that were going in to the villages to give food aid. And they would also end up posing [for pictures] with the indigenous community with close contact and all the [public health] SOPs [standard operating procedures] not being followed. So there was a big dilemma … do you really need to have that picture for your Facebook or your social media, while at the same time, endanger or possibly, in fact, bring infection [to the village]? In the indigenous villages, if one person gets it in the village, there’s no such thing as social distancing, everybody will get it because of the way of life…. In the first year, from our records, I think there were no more than six OA [Orang Asli] who were infected with COVID-19…. And all these six got it from towns or from their workplace. And if they live in urban or semi-urban areas and individual houses, it is easy to isolate infected people … but if it's in a traditional rural village setting, it's going to be a problem.

Vulnerable groups also criticized aid providers for sometimes only providing one-off programs which tended to create dependency on aid, but did not build on the existing capability of the community to do more and be more resilient. Some NGOs were also accused of caring more about social media popularity than actual work.

Some NGOs are building NGOs, or they're running NGOs, just to do the charity, or just to do the welfare. And mostly just want this to do one-off programs. There are many popular NGOs, they will have artists, singers as ambassadors … they will go to the ground and they will sell in their Facebook posts or other social media posts, more on sentiment, more on the sad story of the beneficiaries…. The last time I saw this was during this earlier lockdown, there was one female actor came from Peninsular Malaysia. She distributed the food but in front of the [stateless] people who were begging for food…. So then netizens will say “pity them, they have no food”. But if you distribute it properly, if you tell them early, they will come by groups … according to the time that we gave. We educate them to follow instructions and also to be patient in taking food packs, because they are not animals that we just give food [to] right away. The way some NGOs do this is that they sell stories of marginalized communities, of beneficiaries being unethical, sentiments, sad stories, without uplifting their capabilities…. The other point is they didn't highlight the real issue of why [they] are not getting help from the government or other things – for example, why are they are living on the sea? Because of that, people will see that these particular communities just live on food baskets … without thinking about how to uplift or how to grow the community by making them more sustainable. It is easier for NGOs to do one-off programs, compared to thinking about one program that can sustain the community…. So I can say they are unethical, because they just come and go, they post sentiments about beneficiaries without highlighting the real issues…. that they are stateless or undocumented. They cannot get education. The people who don't live here don't have a proper idea. We don’t get the picture. So that's how I think leads to the judgemental and prejudice parts. Do we Malaysians not take it as an issue that the government has to be responsible for?

In summary, the circle of maleficence and non-beneficence in Figure 2 allows us to identify the sources of ethical violations in the provision of aid, and also gives us the ability to discuss how aid providers can improve upon their processes during crises to better reach and serve vulnerable groups. We discuss some of these ways in the next section.

## Discussion and conclusion

Through a frequency analysis of emergent themes, we found that beneficence occurred most frequently, with almost 60% of excerpts exhibiting this theme. To some extent, this was expected, with our interviewees being mainly aid providers whose mission was to help vulnerable groups. However, we also saw significant evidence of injustice, lack of autonomy and non-beneficence – with each theme recorded in approximately a third of the data. From the frequency analysis, we conclude that the distribution of humanitarian aid during the first MCO either involved, perpetuated, created or exacerbated many ethical problems on the ground.

The question is this: were these ethical issues caused by and specific to the disruptive and destructive nature of COVID-19, or did the pandemic simply exacerbate underlying persistent issues pertaining to the distribution of aid? To answer this question, we needed to understand the relationships between the ethical themes, as found using HCA and plotted on a graph using MDS. We saw that non-beneficence (i.e., negative beneficence) and maleficence (i.e., negative non-maleficence) almost always co-occurred. We then recontextualized this data to understand the situations in which these ethical problems co-occurred, and the agents responsible for causing these problems. 

What we found was that many of the ethical issues uncovered revolved around systemic, long-standing and foundational issues. In Figure 2, we identified “agents of maleficence and non-beneficence”, i.e., actors who repeatedly appeared in the dataset, who either inflicted harm (such as government and aid providers) or who were reportedly harmed as a result (the vulnerable groups). We further broke down the “government” – not as a singular actor but into relevant governmental agents perpetrating harm, including but not limited to the police, the healthcare system, policymakers and politicians (see Table 3).

The findings lead us to conclude the following points. First of all is the need for stronger ethical practices by government agencies and aid providers, in turn rooted in human rights principles. Despite the best intentions of aid providers, as shown in the high prevalence of beneficence, the co-occurrence of non-beneficence and maleficence highlights the gap in knowledge and skills amongst aid providers, in terms of ethical practices or standards in providing aid to vulnerable populations. This gap can cause more harm to the beneficiaries as these unethical, dehumanizing practices are usually underreported and brushed aside, since the beneficiaries have less social capital than the aid providers. Hence, it is the responsibility of aid providers (NGOs or government agencies) to review existing practices, re-examine their relationships and ways of working with vulnerable populations while adopting more ethical practices grounded in human rights. For example, how intersectional and participatory are their approaches in planning and distributing aid to vulnerable populations?

Second is the importance of increasing unrestricted funding directly to humanitarian aid providers. Typically, these providers have existing relationships with communities and a number have already done significant community mapping to understand the specific needs of communities and households. Thus, increasing the amount of unrestricted, private donations to aid providers is a more efficient mechanism than donating funds through larger channels. The government should consider allocating emergency funding directly to aid providers such as small, grassroots NGOs or community-led organisations to ensure that vulnerable populations are receiving aid indiscriminately. The findings also show that one of the root causes for many unethical practices by aid providers – such as taking pictures of beneficiaries, forcing beneficiaries to share personal information (e.g., identity card numbers), or choosing the type of food aid without consulting the community – stems from the restricted nature of funding and excessive need for reporting for by private donors or companies which allocate corporate social responsibility (CSR) funds. Therefore, there is a need for corporations and private donors to review and re-examine their practices and roles, which may inflict harm on vulnerable populations despite their well-placed intentions.

Thirdly, worrying trends and behaviours of agents of harm (e.g., aid providers, the police, the healthcare system) suggest the overdue need for very large institutional reforms of the provision of aid and the treatment of vulnerable populations. We find that institutions built to protect certain marginalized subgroups of the Malaysian population have systematically failed them over the years, culminating in a negative cascade of events caused by COVID-19. Many vulnerable groups have been harmed by these institutions through discriminatory welfare policies, increased police harassment, dehumanizing immigration raids and the detention of vulnerable populations in overcrowded and unsanitary detention centers. The increased lack in trust in authority figures has not only weakened our ability to contain a public health crisis but also lowers Malaysia’s credibility and reputation in remaining committed to its human rights obligations (e.g., the Universal Periodic Review) as well as upholding the fundamental liberties enshrined in the Federal Constitution. In order to progress and build resilience in managing future health and economic crises, the nation will need to take a deep look at its wrongdoings to figure out how to resolve these existing vulnerabilities

## Data availability

### Underlying data

The transcripts underlying the paper are stored in a confidential, secure location due to the sensitive nature of its contents. Most participants in this study were either undocumented or worked closely with undocumented people, who are often at odds with the local law enforcement and are at a heightened risk for arrests, abuse, and deportations. We are willing to share the transcripts in full with anyone who sends us an email request, but in order to protect anonymity and the confidentiality of our participants, cannot store it in an open data repository. Any individuals who are interested in requesting access to the dataset can do so by explaining the reasons why in an email to the corresponding author to the study, M.N. at her email address: melati@mit.edu. Permission will be granted on a case-by-case basis, and data will be anonymized by removing any identifying information of participants before provided.
