# Supplementary material for: Understanding the impact of the COVID-19 outbreak on vulnerable populations in Malaysia through an ethical lens: A study of non-state actors involved in aid distribution
Source: Wellcome Open Res. 2022 Jan 14;6:263. Originally published 2021 Oct 12. [Version 2] doi: 10.12688/wellcomeopenres.17239.2 (PMC8785141; doi:10.12688/wellcomeopenres.17239.2)
Supplement: Peer review history

# Peer review: Reviewer response for version 2

**DOI:** 10.21956/wellcomeopenres.19471.r48044

**Published:** 2022-01-21

**Authors:** AKM Ahsan Ullah

**Affiliations:** 1 Faculty of Arts and Social Sciences, University of Brunei Darussalam, Gadong, Brunei

I have no further comments to make.

Is the work clearly and accurately presented and does it cite the current literature?

Yes

If applicable, is the statistical analysis and its interpretation appropriate?

Not applicable

Are all the source data underlying the results available to ensure full reproducibility?

No source data required

Is the study design appropriate and is the work technically sound?

Yes

Are the conclusions drawn adequately supported by the results?

Yes

Are sufficient details of methods and analysis provided to allow replication by others?

Partly

Reviewer Expertise:

Migration, refugees, development and health policy

I confirm that I have read this submission and believe that I have an appropriate level of expertise to confirm that it is of an acceptable scientific standard.

# Peer review: Reviewer response for version 1

**DOI:** 10.21956/wellcomeopenres.19053.r46645

**Published:** 2021-12-13

**Authors:** AKM Ahsan Ullah

**Affiliations:** 1 Faculty of Arts and Social Sciences, University of Brunei Darussalam, Gadong, Brunei

During the COVID-19 outbreak, the authors endeavored to look into the NGOs and organizations involved in aid delivery among Malaysia's vulnerable population. Given that Malaysia is one of the worst-affected countries by the pandemic in Southeast Asia, this study has important implications. Without a doubt, the article is well written. However, I have a few observations. My observations are here.

 The vulnerable population is at the heart of the article. It is critical that the authors give the readers a clear understanding of who these people are. According to the author, "in Malaysia, approximately 100,086 cases (68 percent of total confirmed COVID-19 cases) from workplace clusters ^1^ were foreign nationals, the majority of whom were migrant workers working in the manufacturing sector – who were known to be a vulnerable population," implying that these migrant workers are vulnerable. The question now is, what are these migrants vulnerable to?

 When investigating the aid engagement of NGOs and organizations, it is critical to understand which NGOs and organizations the authors are referring to. The paper calls for a better understanding of them (NGOs and organizations).

 In terms of methodology: It is unclear if or not any NGOs or groups will be present. If not, why not? It is also critical that the authors clearly explain to the readers how the participants were chosen and approached. By convenience or by snowballing?

- How did the original target sample size of 40–45 people come about? And why is that?
- Who are the 34 people who were finally chosen as respondents? Is there a profile?

As the paper deals with the impact of COVID-19 on the vulnerable population in Malaysia, I would recommend that the authors examine this article for review: Locked up under lockdown: The COVID-19 pandemic and the migrant population, Ullah *et al*., 2021 ^1^ as it offers first-hand information on how the migrant population globally, socially in Southeast Asia has been impacted directly by the restrictions related to the COVID-19 pandemic. This article is based on research conducted in April 2020 (immediately after the outbreak) implying that the recommended article adds a lot of scholarly value to the article.

 I strongly suggest that the article be indexed after the issues identified have been addressed.

Is the work clearly and accurately presented and does it cite the current literature?

Yes

If applicable, is the statistical analysis and its interpretation appropriate?

Not applicable

Are all the source data underlying the results available to ensure full reproducibility?

No source data required

Is the study design appropriate and is the work technically sound?

Yes

Are the conclusions drawn adequately supported by the results?

Yes

Are sufficient details of methods and analysis provided to allow replication by others?

Partly

Reviewer Expertise:

Migration, refugees, development and health policy

I confirm that I have read this submission and believe that I have an appropriate level of expertise to confirm that it is of an acceptable scientific standard, however I have significant reservations, as outlined above.

## References

[1] : Locked up under lockdown: The COVID-19 pandemic and the migrant population. *Soc Sci Humanit Open* .2021;3(1) : 10.1016/j.ssaho.2021.100126 100126 10.1016/j.ssaho.2021.100126 34173510PMC7985246

# Response

**Published:** 2022-01-11

**Authors:** Melati Nungsari

**Affiliations:** Massachusetts Institute of Technology, USA

*Dear Reviewer,*

*First of all, thank you very much for your constructive comments in helping make our manuscript better. We truly appreciate them and have addressed all of your suggestions below. Our responses are in italics. We hope that the restructuring, changes, and additions in the papers cited have more than adequately addressed your reservations for our paper.*

*Thank you again,*

*Authors*

 During the COVID-19 outbreak, the authors endeavored to look into the NGOs and organizations involved in aid delivery among Malaysia's vulnerable population. Given that Malaysia is one of the worst-affected countries by the pandemic in Southeast Asia, this study has important implications. Without a doubt, the article is well written. However, I have a few observations. My observations are here.

 The vulnerable population is at the heart of the article. It is critical that the authors give the readers a clear understanding of who these people are. According to the author, "in Malaysia, approximately 100,086 cases (68 percent of total confirmed COVID-19 cases) from workplace clusters ^1^ were foreign nationals, the majority of whom were migrant workers working in the manufacturing sector – who were known to be a vulnerable population," implying that these migrant workers are vulnerable. The question now is, what are these migrants vulnerable to?

*Thank you for the inputs. This was elaborated in the later section of the research background in order to keep the introduction brief and concise.*

 When investigating the aid engagement of NGOs and organizations, it is critical to understand which NGOs and organizations the authors are referring to. The paper calls for a better understanding of them (NGOs and organizations).

*Thank you for your inputs. We have revised the paper accordingly under the participant sampling and selection section.*

 In terms of methodology: It is unclear if or not any NGOs or groups will be present. If not, why not? It is also critical that the authors clearly explain to the readers how the participants were chosen and approached. By convenience or by snowballing?

*Yes, NGOs are present. Table 1 details the interviewee profiles, whether they are individuals from NGOs or vulnerable populations. We have also expanded on how the participants were chosen and approached in the participant sampling and selection section.*

- How did the original target sample size of 40–45 people come about? And why is that?

*It was based on the estimation of about 5-7 participants for each category of targeted vulnerable populations. This estimate was based on the capacity we have given the time constraint to conduct qualitative interviews during the lockdown. It was also based on our past experience of collecting data through qualitative interviews where our main objective is to understand in-depth the experiences of vulnerable populations rather than achieving representation of the population.*

- Who are the 34 people who were finally chosen as respondents? Is there a profile?

*Yes, they are listed in Table 1.*

- As the paper deals with the impact of COVID-19 on the vulnerable population in Malaysia, I would recommend that the authors examine this article for review: Locked up under lockdown: The COVID-19 pandemic and the migrant population, Ullah *et al*., 2021 ^1^ as it offers first-hand information on how the migrant population globally, socially in Southeast Asia has been impacted directly by the restrictions related to the COVID-19 pandemic. This article is based on research conducted in April 2020 (immediately after the outbreak) implying that the recommended article adds a lot of scholarly value to the article.

*Thank you for the suggestion. We have cited the paper and found it highly useful to our study.*

 I strongly suggest that the article be indexed after the issues identified have been addressed.

# Peer review: Reviewer response for version 1

**DOI:** 10.21956/wellcomeopenres.19053.r46398

**Published:** 2021-12-01

**Authors:** Andika Wahab

**Affiliations:** 1 Institute of Malaysian and International Studies (IKMAS), The National University of Malaysia, Bangi, Malaysia

This is a well-written article, reflective, convincing, and timely. Some inputs for clarification and considerations from the authors as they further develop and improvise the article.

1. Current title: "Understanding the impact of the COVID-19 outbreak on vulnerable populations in Malaysia through an ethical lens: A study of NGOs and organizations involved in aid distribution" - Comment: The terms used here "NGOs and organisations" are not adequately conclusive/reflective to the actual interviewees. For example, how about individuals as interviewees in the study? Another term the authors may consider using is "non-state actors".
2. Different segments of vulnerable populations (especially non-citizens) have different sets of risks and vulnerabilities. For example, migrant workers with valid employment passes may not have issues coming forward to get vaccinated, but those without employment passes may be afraid to come forward to get vaccinated or access social assistance. Another example, refugees with UNHCR cards may feel confident cooperating with NGOs/UNHCR or other individuals providing social assistance, but those without such recognition may not feel comfortable and secure to come forward. These are not clearly articulated and/or differentiated.
3. The intersection between ethical practices and human rights is not adequately linked. The intersection is more than doing “no harm” in delivering services to the vulnerable populations, as claimed repeatedly by the authors. I am particularly interested to learn, for example, how does the implementation of ethical practices promote and protect human rights; in what areas of human rights are affected, positive or negatively (e.g., the right to health, right to life, right to social security); and what the consequences are.
4. The authors mentioned the importance of institutional reforms, including in the final section (i.e., discussion and conclusion). While the authors indicate the reforms needed (e.g., the need to eliminate discriminatory welfare), the earlier sections (including review of literature) do not adequately explain areas (e.g., policy areas/institutions) needing ‘reforms’ and why.
5. Refer to the second point of discussion (see final section) – while the importance of increasing ‘unrestricted’ funding is justified, the authors do not sufficiently explain what ‘unrestricted’ funding is referred to (i.e., beyond taking pictures on the ground and reporting requirements). The authors may further explain and prioritize the key elements of ‘unrestricted funding’, for example in field reporting and submission of evidence, etc. I personally think that there is no such thing as ‘absolute unrestricted funding’ because this may also lead to a lack of transparency and accountability especially on the part of donors. "Unrestricted funding" - I suppose that the authors should find/explain the minimum thresholds that are acceptable, feasible, and justified - meaning in compliance or promote the human rights principles in aid programmes and funding provision.
6. Refer to the third point of discussion when the authors are quoted writing: “the increased lack in trust in authority figures has not only weakened our ability to contain a public health crisis but also lowers Malaysia’s credibility and reputation in remaining committed to its human rights obligations (e.g., the Universal Periodic Review) as well as upholding the fundamental liberties enshrined in the Federal Constitution” - Comment: The UPR is not a human rights obligation, but a process to monitor human rights obligations of the states. The authors may change the UPR to human rights treaties (i.e., treaties Malaysia has ratified).

Note: The above are just additional points for the consideration of the authors, without them, the article still deserves indexing as it contributes immensely to the existing knowledge corpus and policy appraisals in the field.

Is the work clearly and accurately presented and does it cite the current literature?

Yes

If applicable, is the statistical analysis and its interpretation appropriate?

Yes

Are all the source data underlying the results available to ensure full reproducibility?

Yes

Is the study design appropriate and is the work technically sound?

Yes

Are the conclusions drawn adequately supported by the results?

Yes

Are sufficient details of methods and analysis provided to allow replication by others?

Yes

Reviewer Expertise:

International Migration

I confirm that I have read this submission and believe that I have an appropriate level of expertise to confirm that it is of an acceptable scientific standard.

# Response

**Published:** 2022-01-11

**Authors:** Melati Nungsari

**Affiliations:** Massachusetts Institute of Technology, USA

*Dear Reviewer,*

*First of all, we'd like to thank you for your constructive comments in helping better our manuscript. We've addressed them in detail below, in italics. Thank you again and we hope that the changes we have made have addressed all of your concerns.*

*Authors*

 This is a well-written article, reflective, convincing, and timely. Some inputs for clarification and considerations from the authors as they further develop and improvise the article.

1. Current title: "Understanding the impact of the COVID-19 outbreak on vulnerable populations in Malaysia through an ethical lens: A study of NGOs and organizations involved in aid distribution" - Comment: The terms used here "NGOs and organisations" are not adequately conclusive/reflective to the actual interviewees. For example, how about individuals as interviewees in the study? Another term the authors may consider using is "non-state actors".

*Thank you for the suggestion. We have incorporated those changes.  *

1. Different segments of vulnerable populations (especially non-citizens) have different sets of risks and vulnerabilities. For example, migrant workers with valid employment passes may not have issues coming forward to get vaccinated, but those without employment passes may be afraid to come forward to get vaccinated or access social assistance. Another example, refugees with UNHCR cards may feel confident cooperating with NGOs/UNHCR or other individuals providing social assistance, but those without such recognition may not feel comfortable and secure to come forward. These are not clearly articulated and/or differentiated.

*We agree with that and have elaborated more in the Research Background section. We had expanded more on this point in the "Recontextualization by Population" section. However, we do not specifically differentiate migrant workers and refugees in our analysis as we did not collect such data from the participants (the question on the legal status is avoided in the interview as it is deemed quite sensitive given our past experience interacting with migrants and refugees).*

1. The intersection between ethical practices and human rights is not adequately linked. The intersection is more than doing “no harm” in delivering services to the vulnerable populations, as claimed repeatedly by the authors. I am particularly interested to learn, for example, how does the implementation of ethical practices promote and protect human rights; in what areas of human rights are affected, positive or negatively (e.g., the right to health, right to life, right to social security); and what the consequences are.

*You are correct. In fact, the Universal Declaration on Bioethics and Human Rights published by UNESCO in 2006 is grounded on these two fields -- bioethics and human rights. In the literature, there is also growing attention on the link between human rights and bioethics. Ashcroft (2010), for instance, discussed the rising discourse of bioethics being subsumed under human rights practice. This topic while being important is another subject that we could not do justice to in this article.*

1. The authors mentioned the importance of institutional reforms, including in the final section (i.e., discussion and conclusion). While the authors indicate the reforms needed (e.g., the need to eliminate discriminatory welfare), the earlier sections (including review of literature) do not adequately explain areas (e.g., policy areas/institutions) needing ‘reforms’ and why.

*Thank you for your inputs. The conclusion on the need for institutional reform is based on the findings summarised in Table 3 and Figure 2 (p. 13-14). We have also expanded on this part to specify areas needing reforms.*

1. Refer to the second point of discussion (see final section) – while the importance of increasing ‘unrestricted’ funding is justified, the authors do not sufficiently explain what ‘unrestricted’ funding is referred to (i.e., beyond taking pictures on the ground and reporting requirements). The authors may further explain and prioritize the key elements of ‘unrestricted funding’, for example in field reporting and submission of evidence, etc. I personally think that there is no such thing as ‘absolute unrestricted funding’ because this may also lead to a lack of transparency and accountability especially on the part of donors. "Unrestricted funding" - I suppose that the authors should find/explain the minimum thresholds that are acceptable, feasible, and justified - meaning in compliance or promote the human rights principles in aid programmes and funding provision.

*Thank you for the inputs. We have changed it to ‘less restricted’, instead of ‘unrestricted’ funding.*

1. Refer to the third point of discussion when the authors are quoted writing: “the increased lack in trust in authority figures has not only weakened our ability to contain a public health crisis but also lowers Malaysia’s credibility and reputation in remaining committed to its human rights obligations (e.g., the Universal Periodic Review) as well as upholding the fundamental liberties enshrined in the Federal Constitution” - Comment: The UPR is not a human rights obligation, but a process to monitor human rights obligations of the states. The authors may change the UPR to human rights treaties (i.e., treaties Malaysia has ratified).

*Thank you for the inputs. We have removed the UPR to avoid confusion.*

 Note: The above are just additional points for the consideration of the authors, without them, the article still deserves indexing as it contributes immensely to the existing knowledge corpus and policy appraisals in the field.

***Reference***

*Richard E. Ashcroft, Could Human Rights Supersede Bioethics?, Human Rights Law Review, Volume 10, Issue 4, December 2010, Pages 639–660, https://doi-org.libproxy.mit.edu/10.1093/hrlr/ngq037*
